# Improving access to general practice for and with people with severe and multiple disadvantage: a qualitative study

**DOI:** 10.3399/BJGP.2023.0244

**Published:** 2024-04-03

**Authors:** Lucy C Potter, Tracey Stone, Julie Swede, Florrie Connell, Helen Cramer, Helen McGeown, Maria Carvalho, Jeremy Horwood, Gene Feder, Michelle Farr, Bridging Gaps

**Affiliations:** Centre for Academic Primary Care, Bristol Medical School, University of Bristol, Bristol.; Centre for Academic Primary Care, Bristol Medical School, University of Bristol; National Institute for Health and Care Research Applied Research Collaboration West, University Hospitals Bristol and Weston NHS Foundation Trust, Bristol.; One25, Bristol.; One25, Bristol.; Centre for Academic Primary Care, Bristol Medical School, University of Bristol, Bristol.; Centre for Academic Primary Care, Bristol Medical School, University of Bristol, Bristol.; One25, Bristol.; Centre for Academic Primary Care, Bristol Medical School, University of Bristol; National Institute for Health and Care Research Applied Research Collaboration West, University Hospitals Bristol and Weston NHS Foundation Trust, Bristol.; Centre for Academic Primary Care, Bristol Medical School, University of Bristol, Bristol.; Centre for Academic Primary Care, Bristol Medical School, University of Bristol; National Institute for Health and Care Research Applied Research Collaboration West, University Hospitals Bristol and Weston NHS Foundation Trust, Bristol.

**Keywords:** access to health care, continuity of care, general practice, health inequalities, service organisation, women’s health

## Abstract

**Background:**

People with severe and multiple disadvantage (SMD) who experience combinations of homelessness, substance misuse, violence, abuse, and poor mental health have high health needs and poor access to primary care.

**Aim:**

To improve access to general practice for people with SMD by facilitating collaborative service improvement meetings between healthcare staff, people with lived experience of SMD, and those who support them; participants were then interviewed about this work.

**Design and setting:**

The Bridging Gaps group is a collaboration between healthcare staff, researchers, women with lived experience of SMD, and a charity that supports them in a UK city. A project was co-produced by the Bridging Gaps group to improve access to general practice for people with SMD, which was further developed with three inner-city general practices.

**Method:**

Nine service improvement meetings were facilitated at three general practices, and six of these were formally observed. Nine practice staff and four women with lived experience of SMD were interviewed. Three women with lived experience of SMD and one staff member who supports them participated in a focus group. Data were analysed inductively and deductively using thematic analysis.

**Results:**

By providing time and funding opportunities to motivated general practice staff and involving participants with lived experience of SMD, service changes were made in an effort to improve access for people with SMD. These included prioritising patients on an inclusion patient list with more flexible access, providing continuity for patients via a care coordinator and micro-team of clinicians, and developing an information-sharing document. The process and outcomes improved connections within and between general practices, support organisations, and people with SMD.

**Conclusion:**

The co-designed strategies described in this study could be adapted locally and evaluated in other areas. Investing in this focused way of working may improve accessibility to health care, health equity, and staff wellbeing.

## Introduction

This study is concerned with access to general practice for people with severe and multiple disadvantage (SMD). It builds on previous work, in which continuity of care, being able to develop a trusting relationship, and being proactive were found to be of particular importance in providing care to people with SMD.^1^^–^5 It also emphasises the importance of making services more trauma-informed; trauma-informed care *‘realizes the widespread impact of trauma and understands potential paths for recovery; recognizes the signs and symptoms of trauma in clients, families, staff, and others involved within the system; and responds by fully integrating knowledge about trauma into policies, procedures, and practices, and seeks to actively resist re-traumatization’*.^6^

**Figure 1.**
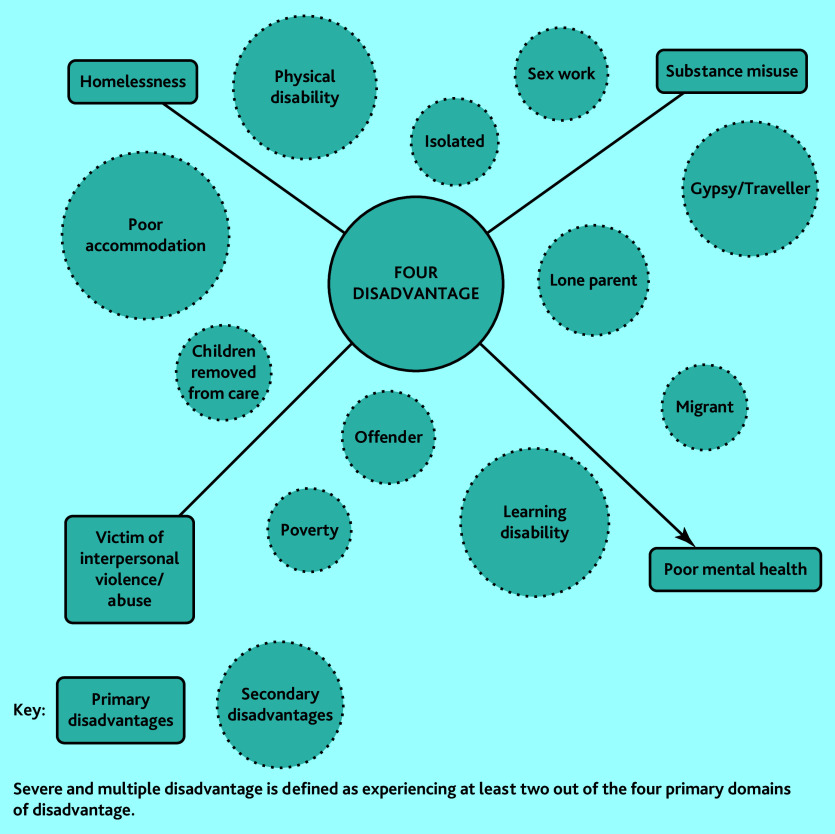
Primary and secondary domains of disadvantage. Adapted from Sosenko *et al*.^7^

SMD is defined here using the gender-sensitive conceptualisation of experiencing at least two out of four primary domains of disadvantage: homelessness, substance misuse, victim of interpersonal violence and abuse, and poor mental health (Figure 1).^7^ In England, 2.3 million adults (5.2% of the population) experience ≥2 of these primary domains in a single year.^7^ The combined and intersecting effect of multiple sources of severe disadvantage carries an extremely high burden of mortality, multimorbidity, and frailty.^8^^–^10 However, people with SMD encounter significant barriers to accessing primary care and lower enablement (the impact of the encounter on patients’ ability to understand and manage their health problems).^4^^,^^11^^,^^12^ People with SMD are more likely to have negative experiences of health care, including stigma and discrimination, which can act as a lasting deterrent to help seeking, and appointment systems are often incompatible with their help-seeking behaviours.^4^^,^^13^ These patients are highly marginalised and most general practice does not effectively include them.^4^^,^^14^^–^16 The concept of access in this study has four key aspects: availability (including direct and indirect costs to the patient), utilisation, service relevance and effectiveness, and equity (the extent to which resources are mobilised to reflect need).^17^ Access to care is more than just being registered at a general practice; it requires the ‘human fit’ between the patient and healthcare staff.^18^ Not being able to provide adequate care to patients who are disadvantaged contributes to GP stress and burnout.^12^^,^^19^ Improving the ‘human fit’ between general practice and those most in need is therefore good for staff as well as patients.

**Table:** How this fits in

This study builds on previous work that found that continuity of care, developing trusting relationships, and being proactive are of particular importance in providing care to people with severe and multiple disadvantage (SMD). This study describes strategies co-designed with service users, including prioritising patients on an inclusion patient list with more flexible access, providing continuity from a care coordinator and micro-team of clinicians, and producing an information-sharing tool, in addition to rich contextual information on how to change ways of working to achieve better care for people with SMD. These strategies are practical examples of proportionate universalism in general practice, where resources are prioritised to those most in need. They could be adapted and piloted in other practices and areas, and may offer promise in improving inclusion of other marginalised groups. Investing in this focused way of working may improve accessibility to health care, health equity, and staff wellbeing.

Specialist homeless healthcare centres have emerged in most major cities in the UK to provide primary care to people experiencing homelessness, but this is only part of the solution. Many people experiencing SMD are not homeless, or may only be homeless temporarily, and certain groups such as street sex workers often do not access homeless services because of safety concerns.^4^ Specialist clinics and outreach are important and useful resources for people in marginalised communities, but they are often separate from mainstream services, unable to offer the full spectrum of mainstream primary health care, and may have limited staffing and opening hours. There are also challenges in supporting people to transition from specialist services into mainstream care as their crisis situations stabilise and health problems improve.^20^ Limited outreach or homeless health specialist provision is not enough; there is a need for mainstream primary care to be more inclusive, integrated, and accessible to people with SMD. General practice is a stretched system in the UK that can be hard for people with SMD to access, with current provision not proportionate to need.^12^

This study used a co-production approach with researchers and stakeholders, including people with lived experience of SMD and healthcare professionals, developing collaborative partnerships, meaning that people with SMD can become more equal partners, sharing decision-making roles.^21^^,^^22^ Co-production may result in more implementable interventions and lead to better outcomes.^23^^–^25 The priorities and abilities of people with SMD and the organisation of health services are poorly aligned and inequalities in access, quality, and outcomes in care are worsening,^26^ creating and exacerbating vulnerabilities.^27^ Co-production involving people with lived experience of SMD and general practice staff offers an opportunity to challenge this discord and bring together people with SMD and health services.

The aim of this research was to improve access to primary care for people with SMD by collaborating with people with lived experience of SMD, a charity that supports them, and general practice staff. This study sought to articulate the perspectives of people with SMD, those who support them, and general practice staff participants about their experience of co-producing service improvements and improved access. The research focused on the answers to two practical questions: what are the key issues and challenges in improving access to general practice for people with SMD; and what are the potential strategies to improve access to general practice for people with SMD?

## Method

Development of the co-production project is described in Box 1. The second phase of the project, described in the present study, ran between July 2021 and August 2022. Project methods were informed by participatory action research and cooperative inquiry, where research is co-developed with, rather than on, people.^30^^,^^31^ One researcher, who is an experienced, non-clinical, qualitative researcher with no previous involvement with the group but a background of working with marginalised populations and healthcare professionals, joined the project to conduct interviews, observations, and second coding to provide a more independent perspective. For the comfort of participants with lived experience of SMD, where possible, a choice of interviewer was offered.

**Box 1.** Co-production group project development

The co-production group (called Bridging Gaps) was started in May 2019 by the lead author and women with lived experience of SMD who had been supported by One25, a Bristol charity that aims to support some of the city’s most marginalised women. As a GP, the lead author had delivered an outreach clinic once a week at the drop-in centre of One25 for 2 years before the start of the project and had therefore built up a number of trusted relationships. One25 is a women-only safe space, and the lived experience team decided to continue the co-production group as a women-only safe space while improvement work might focus on different aspects of access for all people with SMD. Co-production meetings were held every 2 weeks and took place in community spaces. Participants were offered shopping vouchers as thanks for their time. We provide a detailed account of our co-production experiences elsewhere.^28^^,^^29^
After team building, general practices identified in areas with higher concentrations of people with SMD were contacted. Face-to-face sessions were held with staff in two general practices and one with the GP training scheme. Five online sessions were held with staff in general practices during the COVID-19 pandemic. When possible, the group opted to resume face-to-face collaborative work with staff in general practices. Three service improvement meetings were held at each of three GP practices (nine in total); this second phase of GP service improvement meetings is the main focus of the present study.

Workers from One25, a charity that aims to support some of Bristol’s most marginalised women, helped to recruit and support participants with lived experience of SMD to the co-production group.^29^ Participants with lived experience of SMD were separately offered opportunities and informed decision-making processes for being a lived experience collaborator on the project, for being a research participant (governed by ethics approvals), and/or for being a co-author, respecting International Committee of Medical Journal Editors recommendations.^32^ Individual members of the co-production group could, and did, make different informed decisions on each of these opportunities based on what was right for them, with the support of a support worker.

Three service improvement meetings were facilitated at three general practices (nine in total), and included members of the co-production team, a support worker from One25, researchers, and selected staff from the general practice. The first meeting at each practice introduced access to general practice for people with SMD and participants shared their experiences and perspectives, while subsequent meetings focused on co-designing plans to improve access. The nine service improvement meetings were held between November 2021 and June 2022. Data collection comprised semi-structured interviews with four participants with lived experience of SMD before the service improvement meetings (July to November 2021); observations of six of the nine collaborative service improvement meetings (12 hours in total with 22 participants), with documentary analysis of meeting minutes of the remainder; semi-structured interviews with nine GP staff (June to August 2022); and a focus group with three participants with lived experience of SMD and one staff member from One25 (July 2022). Participants in the service improvement meetings were offered the opportunity to be interviewed or be part of the focus group. In total, 30 participants from a range of roles contributed to this phase of the study, including 14 participants in the co-production team and 16 primary care staff (Table 1).

**Table 1.** Participant roles

**Role**	**Participants, *n***	**Category used in quotations**
**Co-production team (Bridging Gaps group)**		
Patient with lived experience of SMD	8	Bridging Gaps (BGW)
Support worker	2	Partner organisation (Ptn)
Academic GP	2	Researcher (Res)
Researcher	2	Researcher (Res)

**General practice participants**		
GP	6	General practitioner (GP)
Nurse	2	Nurse (Nurse)
Drug and alcohol worker	3	Drug and alcohol (D&A)
Care coordinator/link worker/social prescriber	5	Care coordinator (CC)

**Total**	**30**	

*SMD = severe and multiple disadvantage.*

### Data analysis

The focus group and interviews were audio-recorded and fully transcribed. Meeting observation notes and interview/focus group transcriptions were coded using NVivo (version 1.6.1) software. The first two transcripts were coded independently and discussed to explore differences in interpretation and maximise rigour. A mix of inductive and deductive (based on project aims) coding were used and the data were collated into themes. Data were analysed using reflexive thematic analysis,^33^ with several theme development meetings with other researchers. Overarching themes were developed by transferring between visual mind maps, narrative text, and discussions with the research team.

Several authors had been involved in the co-production team for 2–3 years, and over time the participants with lived experience of SMD became colleagues. Reflexivity was essential in harnessing the value of the in-depth involvement of personal experience in this work. Exploring access to general practice for people with SMD while challenging power relations is complex; reflexive thematic analysis allowed the flexibility to follow an iterative path in order to better understand this. The close involvement of several of the research team, including facilitating collaborative meetings, meant they were highly familiar with the data and enabled critical engagement throughout.

## Results

**Figure 2.**
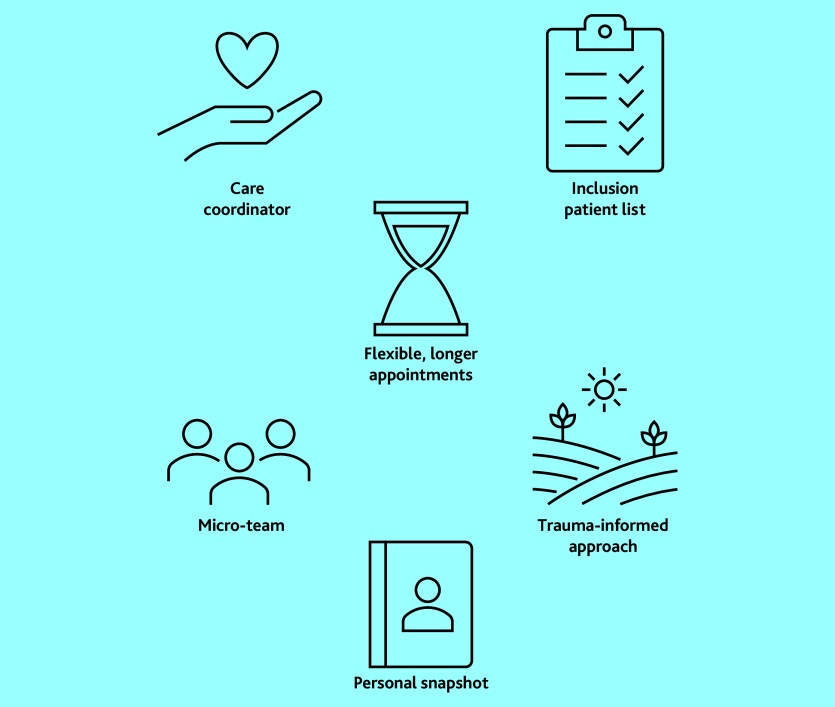
Co-designed general practice service changes.

Several service changes to improve access and care for people with SMD were collaboratively developed and implemented in participating practices. These are summarised in Table 2 and Figure 2. Participants with lived experience of SMD and healthcare professionals shared their perspectives on these changes, their experiences of collaborating to develop them, and their opinions on how best to provide care for people with SMD. These findings are presented below as three main themes, which were developed from the data, illustrated with anonymised verbatim quotes:

- time and funding opportunities enabled motivated individuals and teams to improve general practice with people with SMD;
- participants shifted ways of working to provide proactive continuity of care from trusted healthcare professionals; and
- improving connections within and between general practice, support organisations, and people with SMD was enjoyable, encouraged empathy, and was beneficial to patients and staff.

**Table 2.** System problems and changes implemented to improve access and care for people with SMD

**System problem**	**Change implemented**	**Illustrative quotes**
Healthcare professionals and participants with lived experience of SMD highlighted that people with SMD often fall through the gaps of mainstream general practice	**1. Care coordinator and patient list** Two mainstream practices independently used the opportunity of care coordinator funding to provide an individual or team for additional support for people with SMD at their practices. Both practices have continued these roles beyond the project and one is recruiting for a second care coordinator because of high levels of need. Participants recognised the value in these individuals maintaining and advocating for a list of patients with SMD. Care coordinators proactively communicate with patients on the list (without ‘nagging’), provide continuity, and help clinicians to provide appropriate care.	*‘The role that* [CC1] *has is developing into something that’s quite essential to the practice for this particular group of people with complex needs, and not only them, but for the staff … it gives us somewhere to put our thinking in terms of how we behave with people with complexity, people we can’t get hold of, certain types of vulnerability.’* (GP2) *‘The doctors will send me tasks and say, “This one needs to be on your radar.”’* (CC1)
Mainstream appointment systems are often inaccessible to people with SMD who often require rapidly responsive, easily accessible routes into care	**2. Prioritise flexible access and longer appointments to patients in greater need** One practice dovetailed the care coordinator role with a specialist inclusion health clinic for people with SMD; in another the care coordinator had a direct telephone line for patients with SMD and priority access to appointments when needed. One practice also had protected appointment slots that could be used by drug and alcohol workers for one of their clients if needed.	*‘I’ve also got the authority, the clearance, to book appointments in when I need to, whereas the staff, they’d have to say, “Okay, we haven’t got any more for today and the next one’s in two weeks.” I can change a slot with the doctor.’* (CC1)
Several participants reflected on the variability in behaviour or prejudice from different staff members and this was a deterrent as they wouldn’t know if they would get ‘a nice one’	**3. Trauma-informed approach throughout the whole practice in addition to micro-team of staff focused on people with SMD** Both of the mainstream general practices involved in the project requested training for all their staff and reflected on the importance of a whole-team approach to improving access for people with SMD. This trauma-informed training could not be provided within the resources of this study; however, it is being planned through other local funds. Additionally, it was valuable to have a micro-team made up of clinicians and care coordinators who were able to provide continuity to patients and so promote trust. One care coordinator offered patients on her list a direct telephone number that only she would answer so patients had the security of knowing who they would speak to.	*‘So I think a lot of my barriers was around the way I’d been treated in GP surgeries before. Like I said, not about the GPs, but the receptionists.’* (BGW2) *‘That first meeting was a whole-team meeting because I do feel like access to health care is very much a whole-team issue. It’s not just about GPs.’* (GP1)
Participants with lived experience of SMD raised the need to re-tell their story to multiple staff members as a barrier to accessing general practice	**4. Personal snapshot document as a tool to share information with general practice (see Supplementary Information S1)** To try to mitigate this, participants co-developed a personal snapshot document to share information with the general practice, which is completed with a support worker and emailed securely to the practice, with an alert on electronic notes to see the document before consultation. It is optional, but can include information that individuals may want to share with the practice, whether they would like to be asked about these or not, and best ways of working with them. This uses a trusted connection with a support worker to help build a relationship with general practice staff.	*‘There’s so many services that support people but so few of them are actually able to support people in* [general practice] *… if you’ve got one good relationship and somebody can just do that with one person then they’ve got a much higher chance of being able to communicate what they need.’* (Ptn2) *‘It’s really helpful to the GPs to have the background before doing consultations with patients and it gives the patient more confidence and trust in us that we’ve listened to them and adjusted things to help them.’* (CC1)
Participants raised serious concerns about women not feeling safe to access homeless health or drug and alcohol services as the locations are targeted by perpetrators of violence and abuse	**5. Exploring potential changes including providing healthcare outreach clinics in women-only safe spaces** Participants discussed ideas of collaborating with women-only safe spaces in the city, such as women-only hostels, or locations that were not focused on drug use or homelessness to host a regular clinic for an outreach clinician to support women with SMD. Healthcare staff who work with people experiencing homelessness were enthusiastic to support this; however, funding has not yet been secured.	*‘It’s hard to get normality into your life, access accommodation services, when these people* [men] *are there. The idea of ‘drop in’ is great but there are all these blokes outside.’* (BGW2)

*SMD = severe and multiple disadvantage.*

### Time and funding opportunities enabled motivated individuals and teams to improve general practice with people with SMD

Participants with lived experience of SMD and general practice participants shared their experiences of barriers to access for people with SMD. All participants expressed motivation to tackle these barriers. Protected, facilitated meeting time with women with lived experience of SMD galvanised healthcare professionals who were already interested in improving care for people with SMD. General practice staff used Primary Care Network care coordinator and Enhanced Access funding to make service improvements (Table 2), which have continued beyond this project. This included two practices recruiting a care coordinator to support people with SMD who struggle to access mainstream general practice, and one practice co-designing an inclusion health clinic. This clinic included support from the care coordinator with appointment booking, reminders, and non-clinical support, and longer appointments with one of two GPs experienced in inclusion health. This additional capacity enabled motivated GPs and care coordinators to collaborate on providing better care for people with SMD:

*‘So, it was partly GP13 from the partners and partly the local contract funding that fitted together. So then me and another GP with an interest in inclusion health were given a mandate … to crack on with it and so we did. Then Bridging Gaps was there as a really useful opportunity for people with lived experience who were doing relevant work around improved access for people with experience of trauma. So it felt like a very ideal synergy.’*

(GP5)

When GPs were asked what was the hardest part of providing care to people with SMD, the most frequent answer was not enough time. GP participants were conscious that people with SMD had a high level of need and described really wanting to do a good job in providing care for them. Participants had some knowledge of how to do this, but described being limited when simultaneously trying to safely manage high volumes of clinical demand:

*‘If you’re trying to deal with someone with really complex needs in the middle of an absolutely overbooked on-call clinic with fifty calls, and you’re just trying to get through the session safely … you are going to really struggle to provide the empathetic, whole person care that you might want to provide. So, structurally, you need to put clinicians in a place where they’ve got the headspace and the opportunity to be kind and trauma-informed and aware of that person’s needs. Otherwise, it’s just not fair on either person.’*

(GP5)

In considering service improvements to better include people with SMD, some participants recognised the potential for overwhelm or burnout in healthcare professionals who do not have sufficient capacity for additional work, and how that can detriment the compassion people feel able to offer and risk staff leaving:

*‘I think that staff are really tired … they’re very kind of stretched and I think that impacts on people’s compassion … staff burnout is I think quite common at the moment.’*

(Nurse1)

One GP who delivered the inclusion health clinic developed during the project using Enhanced Access funding alongside mainstream general practice highlighted how enjoyable it was to have protected time to do a good job for patients who really need it:

*‘I think as well as hopefully being good for the patients. It’s quite joyful for us, it’s really improved our work.’*

(GP1)

Another GP shared the potential of the inclusion health clinic to improve efficiency and reduce stress elsewhere in the system, as standard clinics are not appropriately timetabled for managing high levels of complexity that could be managed in the inclusion health clinic:

*‘I think, overall, that* [colleagues] *were supportive of* [the inclusion health clinic] *… allowing a space for patients who needed more time where it was really stressful to try and do that within a general clinic with fifteen patients. So hopefully, it offloaded some of the stress.’*

(GP5)

### Participants shifted ways of working to provide proactive continuity of care from trusted healthcare professionals

Several participants with lived experience of SMD described negative experiences, including feeling judged by or discriminated against by authority figures, which were barriers to accessing care. Examples included having to wait longer than other patients if attending the surgery to collect opiate substitution prescriptions, or having this called out publicly in the waiting room, exacerbating feelings of shame around substance dependence. These feelings mattered and persisted; building trust seemed to be key in enabling engagement. Most participants with lived experience of SMD and healthcare professionals highly valued continuity as a strategy to achieve this:

*‘Trust is won in small moments, isn’t it, over a period of time? In terms of structural things that facilitate that … continuity really matters. Yeah, it does, particularly for this group of patients.’*

(GP5)

*‘*[W]*hen you are ready, you’re gonna go to that person ’cause you’ve built that trust along the way. I trust that they’re not gonna railroad me into something.’*

(BGW2)

Some participants referred to the variability of behaviour people with SMD might receive from different staff members, and therefore it was important for them to know who they were going to speak to. A few participants who provided additional support to people with SMD recognised the benefit of building on existing trusted relationships to encourage engagement with other healthcare professionals, or help with the practicalities of getting to appointments:

*‘So we actually get that person to feel that it’s okay, if we’re all singing from the same hymn sheet and saying, “You need to go there. Just ask for this person. You’ll be absolutely fine.”’*

(CC1)

Healthcare professionals who provided enhanced care for people with SMD highlighted the importance of contacting them proactively. They emphasised the importance of not being too forceful and listening to patients’ priorities first before using opportunities to offer assessment or management that might be the healthcare provider’s agenda. Both healthcare professionals and patients valued proactive, person-centred communication, which offers a way to show compassion and a way to build relationships:

*‘I text people a lot … sometimes I’ll just send a text saying, “I hope you’re okay” … especially when people aren’t engaging, just keeping that relationship going and then when you meet up with them, they will sometimes say, “Oh, you know, I like getting your texts, thank you.”’*

(Nurse2)

Participants with lived experience of SMD encouraged general practice staff to remember *‘the quiet ones’* (observation notes), patients who might not actively ask for or advocate for their needs and are often left behind by general practices that require people to actively seek care and overcome common obstacles such as limited appointment slots. This resonated with several practice staff. One GP described the balance between being proactive with ‘the quiet ones’ and respecting patient choice:

*‘Don’t forget the people who are traumatised and go quiet and disappear, but at the same time, we mustn’t hound people … allowing the patient autonomy and control where possible.’*

(GP1)

The importance of honest communication and human connection with people with SMD was highlighted by participants with lived experience of SMD and healthcare professionals, and enabled progress of patient and healthcare professional agendas. One participant with lived experience of SMD reflected on feeling patronised by healthcare professionals in the past, and contrasted this with the open communication used by a care coordinator who collaborated on the project:

*‘As a service user you walk in and I know when they’re putting on a voice … just be yourself, do you know what I mean? … I think CC1 to me seems, when you meet her in the surgery is how she would talk at home, do you know what I mean? It’s just her.’*

(BGW2)

### Improving connections within and between general practices, support organisations, and people with SMD was enjoyable, encouraged empathy, and was beneficial to patients and staff

Both the process of co-production and the service improvements that were developed (Table 2) initiated or strengthened teamwork and connectedness within and between general practice teams. Some participants noted the value of relationships and teams in general practice that were focused on delivering better care for people with SMD. One participant described relief at feeling part of a wider team of practices also motivated to tackle health inequalities:

*‘It’s really kind of encouraging for us to know that there are other GP practices that are kind of passionate about health inclusion; that’s, you know, refreshing and a relief!’*

(Nurse1)

Co-producing service improvements encouraged empathy and human connection, and was rewarding to healthcare professionals and people with SMD. Patients and staff were enthusiastic about the benefits of the changes they had made, as well as outcomes for patients, primary care, and collaborating organisations:

*‘There is people behind these labels and there is humans and human behaviour and I think we have made the difference. I think we made the difference like in* [Practice2]*, I think we made a difference in* [Area8] *… I love that and I think that’s a massive thing.’*

(BGW2)

A couple of participants with lived experience of SMD reflected that their experience of collaborating with general practice staff helped them empathise with healthcare professionals and feel more comfortable and empowered to engage with general practice:

*‘It’s actually kind of broken down my own barriers towards GPs … it’s made me a little bit more confident to speak up and sort of put my view across and know that I’m able to do that; this is just another human being I’m talking to.’*

(BGW1)

General practice participants highlighted the value of meaningful collaboration between those with lived experience of SMD and providers in improving contextual understanding. Collaboration improved empathy, challenged assumptions, enabled those with lived experience of SMD to feel listened to, and increased the relevance of services to the local population. This new way of working had a different balance of power, which general practice participants found insightful and constructive:

*‘When you change the power dynamic and have a meeting like that, where we’re all on an equal footing, I often find that you have unexpected insights … they’ve challenged the way that we’ve thought about things and given us a fresh way of thinking … it’s improved things for us as doctors as well as for our patients.’*

(GP1)

## Discussion

### Summary

This study presents an example of using highly collaborative and inclusive methods to develop sustained service changes to improve access to general practice for people with SMD. Building on the foundation of a highly inclusive co-production project, motivated individuals and teams were enabled to achieve service changes with protected time, funding opportunities, and the involvement of patients with lived experience of SMD. Co-designed service improvements included (Table 2 and Figure 2):

- using care coordinators to hold and advocate for a marginalised patient list;
- prioritising flexible access and longer appointments for patients in greater need;
- promoting a trauma-informed approach throughout the whole practice team in addition to a micro-team of clinicians and care coordinator focused on supporting and providing continuity to people with SMD; and
- developing a personal snapshot document as a tool to share information with the general practice.

With protected time and proactive support from care coordinators, GPs enjoyed ‘doing a good job’ for patients who really needed it. This might also ease pressure on other general practice clinics. In effect, the teams used local funding opportunities to support some of the extra types of GP work seen in areas of deprivation that are typically unrecognised and under-resourced.^19^ The process and outcomes of this work improved connections within and between general practices, support organisations, and people with SMD, which was beneficial to all. Investing in this focused way of working may improve healthcare accessibility, health equity, and staff wellbeing.

### Strengths and limitations

A key strength of this work was the significant involvement of participants with lived experience of SMD and those who work closely with them throughout the study. This provided a refreshing and connecting experience for both general practice and participants with lived experience of SMD, and helped galvanise change focused on what is important to those it is intended to help. The intense involvement of several members of the research team in the project not only offers strength in the depth of understanding of the data and context, but also the potential for bias. Reflexive thematic analysis values the researcher’s subjective experience as part of the analysis process. Having several members of the research team who were detached from the project meant they could challenge potential assumptions and encourage reflexive discussion of the influence of subjective expertise.

A decision was made by the team not to include men in the lived experience co-production team. Marginalisation is more damaging to women than men,^10^ and women who have experienced street sex work/prostitution are often the least well heard of the inclusion health groups;^2^ therefore, prioritising their involvement and safety took priority. The project aimed to improve access to general practice for everyone with SMD and the service changes were not restricted by gender. However, the individuals involved cannot represent the full diversity of opinion of people with SMD. Gender sensitivity is a vital component of trauma-informed care,^6^ and seems to be particularly so in SMD, given that mortality ratios are worse for women than men who experience SMD.^10^

### Comparison with existing literature

There is currently insufficient systematic review evidence to make clear recommendations on how to improve access to primary care.^17^ This study is in keeping with other work showing that proactivity, continuity of care, and being able to build a trusting relationship are of particular importance in providing care to people with SMD,^1^^–^5 but furthers this by demonstrating how this can be achieved in practice alongside other service improvements to improve access for and with people with SMD.

In the UK, GPs experience high stress levels, particularly in having ‘insufficient time to do the job’, with large numbers leaving or considering leaving the profession.^34^ The increased burden of ill health and multimorbidity in socioeconomically deprived areas, alongside fewer GPs per head of need-adjusted population than in affluent areas, results in high demands on primary care and increased GP stress for those in areas of greater socioeconomic deprivation.^12^^,^^35^ There is a risk of experiencing moral distress in health care, which comes about from knowing the right thing to do while being in a situation in which it is nearly impossible to do it.^36^ This study outlines some strategies that can offer hope. Healthcare professional participants, who were given additional resources and time to better care for patients who experience substantial health inequities, were enthusiastic about the process and outcomes for both patient care and the staff team.

With limited resources, there is an imperative to make decisions on the principles of proportionate universalism, that health actions must be with a scale and intensity that is proportionate to the level of disadvantage.^37^ The service improvements co-designed in the current study represent positive selectivism, where targeted approaches that sit alongside universal services are used to cater for specific needs.^38^ The positive selectivism of people with SMD was managed by micro-teams of clinicians in the practices, with the care coordinator holding the patient list and providing proactive connection and advocacy, working alongside a small number of GPs who were experienced and committed to improving care for people with SMD. This technique, working particularly with those who are most committed to making changes for patients, fits with social movement and healthcare improvement literature,^39^ and the literature on primary care micro-teams.^40^ Another study has highlighted the opportunity of support roles such as care coordinators in facilitating timely access to care and embedding relationship-based care into and across routine general practice.^41^ There are similarities with efforts to implement reasonable adjustments for people with intellectual disabilities and/or autism in health care,^42^ and the potential for shared learning in improving inclusion across marginalised groups.

In addition to micro-team focus, all participating practices raised the need for an empathetic trauma-informed approach throughout the whole practice team. People with SMD need to feel safe that any staff member they encounter will treat them respectfully. The experiences shared by participants and the fact that one care coordinator saw the need for a direct phone line that nobody else would answer for patients to feel safe to call suggests that the experience of judgement, stigma, and prejudice are still present in healthcare interactions. This is consistent with other studies,^11^^,^^43^ but having only one trusted staff member is obviously not ideal. While the influence of societal prejudice against addictions, mental ill health, and homelessness, alongside other potentially intersecting biases, are undoubtedly challenging to tackle, there is evidence that staff training can improve trauma-informed knowledge, attitudes, and behaviours.^44^

Healthcare access has recently been described as the ‘human fit’ between the needs and abilities of the population and the abilities and capacity of the healthcare workforce to meet those needs, in the context of particular societal conditions and organisational structures and processes.^18^ The findings of the current study support this description and extend it by highlighting the structural, personal, and relational elements that support improved access to general practice for people with SMD. This highly inclusive work helps us to move towards a better human fit and relationship between general practice and people with SMD.

### Implications for research and practice

As outlined in this study, co-production can bring fresh thinking to complex problems.^23^^–^25 Some general practices or networks may wish to try similar highly inclusive methods of collaborating with local support organisations and people with lived experience of SMD to develop context-specific service improvements. The investment in doing this properly should not be underestimated: the project described in this study has been 4 years in the making and started from already established trusted relationships.^28^^,^^29^ A more feasible strategy might be to use or adapt the strategies developed in this study, as outlined in Table 2, with important contextual understanding contained in the results and discussion. The authors would encourage others to strive for a level of meaningful engagement with people with SMD, perhaps in collaboration with a local support organisation, as part of adapting these interventions locally.

Further research is needed to understand how to achieve trauma-informed care across the whole practice, including what processes and outcomes are important in improving access and care of people with SMD in general practice, and to clarify what works for whom and in what circumstances. The service changes implemented in this project have relevance to each of the four aspects of access availability (including direct and indirect costs to the patient), utilisation, service relevance and effectiveness, and equity (the extent to which resources are mobilised to reflect need).^17^ There is a need for rigorous complex intervention development and evaluation to better understand the impact of such changes on equitable access to primary care proportionate to need.
